# Reversibility of High-Grade Atrioventricular Block with Revascularization in Coronary Artery Disease without Infarction: A Literature Review

**DOI:** 10.1155/2016/1971803

**Published:** 2016-01-26

**Authors:** Rhanderson Cardoso, Carlos E. Alfonso, James O. Coffey

**Affiliations:** Miller School of Medicine, University of Miami, Miami, FL 33136, USA

## Abstract

Complete atrioventricular (AV) block is known to be reversible in some cases of acute inferior wall myocardial infarction (MI). The reversibility of high-grade AV block in non-MI coronary artery disease (CAD), however, is rarely described in the literature. Herein we perform a literature review to assess what is known about the reversibility of high-grade AV block after right coronary artery revascularization in CAD patients who present without an acute MI. To illustrate this phenomenon we describe a case of 2 : 1 AV block associated with unstable angina, in which revascularization resulted in immediate and durable restoration of 1 : 1 AV conduction, thereby obviating the need for permanent pacemaker implantation. The literature review suggests two possible explanations: a vagally mediated response or a mechanism dependent on conduction system ischemia. Due to the limited understanding of AV block reversibility following revascularization in non-acute MI presentations, it remains difficult to reliably predict which patients presenting with high-grade AV block in the absence of MI may have the potential to avoid permanent pacemaker implantation via coronary revascularization. We thus offer this review as a potential starting point for the approach to such patients.

## 1. Introduction

Permanent pacing is the routine treatment for irreversible third-degree and advanced second-degree atrioventricular (AV) block [1, 2]. Although necessary and effective in most patients with such conditions, the implantation of a permanent pacemaker is costly [3, 4] and is associated with significant potential acute complications, including but not limited to infection [5], bleeding [6], hematoma [2], hemothorax [7], lead dislodgement [8], and atrial [9, 10] or ventricular [9, 11] rupture. Moreover, pacemaker leads over time are subject to fracture, insulation break, or recall, which may require lead extraction, a potentially high-risk procedure [12, 13]. It is therefore desirable from both societal [14] and individual [15] patient points of view to thoroughly rule out all reversible causes of bradyarrhythmia before commitment to a permanent device [2]. Reversible causes of high-grade AV block include hypothyroidism [16], hyperthyroidism [17], lymphoma [18], herbal medications [19], chemotherapeutic agents [20], Lyme disease [21], viral myocarditis [22], apical ballooning syndrome [23], and negative chronotropic agents [24].

Acute myocardial infarction (MI), particularly with injury to the inferior wall, is also a well-described cause of reversible AV block [25–27]. Potential reversibility of high-grade AV block in coronary artery disease (CAD) patients without an acute MI, in contrast, is a relatively unexplored concept. Herein, we present a literature review prompted by a case of unexpected postrevascularization reversal of symptomatic AV block.

## 2. Illustrative Case Description

**Figure 1:**
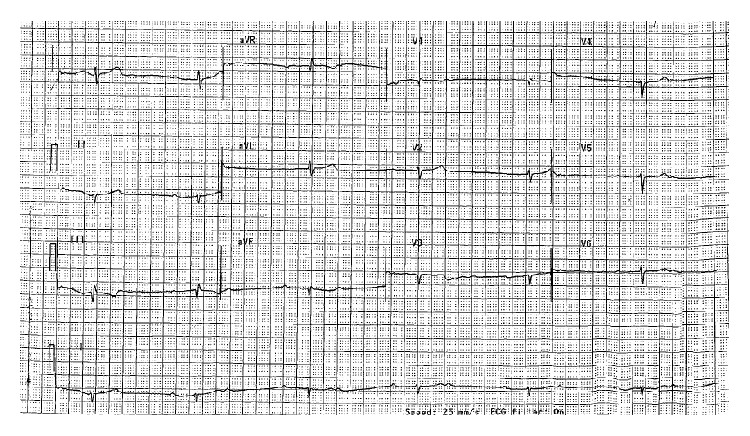
Two : one atrioventricular block in a patient with unstable angina prior to revascularization.

An 85-year-old man with a history of ischemic cardiomyopathy and baseline ejection fraction of 40% following an inferior wall MI in 1996 presented with approximately one week of lightheadedness and chest pain at rest. Previously, his symptoms of chronic stable angina were well controlled with isosorbide mononitrate. An electrocardiogram was performed, revealing 2 : 1 AV block with a ventricular rate of 37 beats per minute (Figure 1). The patient had no prior history of conduction system abnormality. Common causes of reversible AV block were excluded, and the patient was admitted in anticipation of permanent pacemaker implantation. Cardiac biomarkers were negative. Cardiac monitoring continued to show 2 : 1 and occasionally higher-grade AV block.

**Figure 2:**
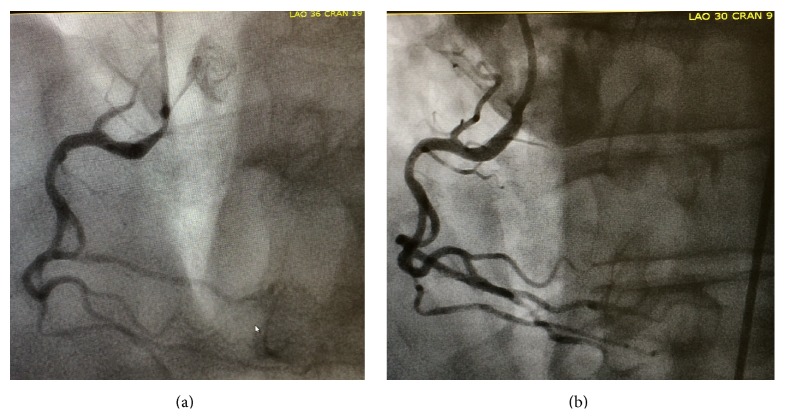
(a) Angiography revealed an 80% ostial stenosis in the right coronary artery; (b) right coronary artery after successful deployment of drug-eluting stent to ostial lesion.

**Figure 3:**
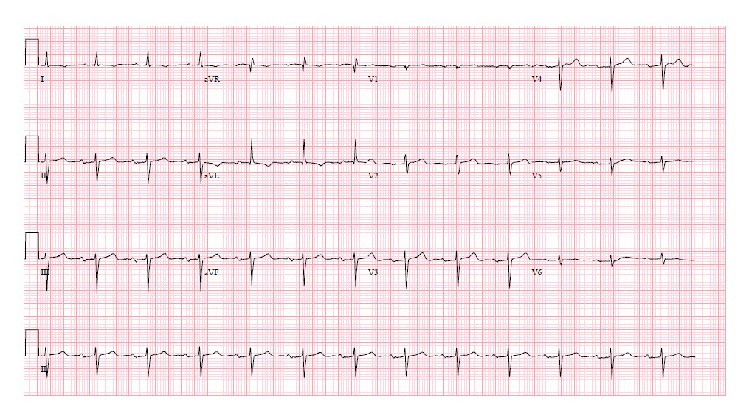
Resolution of 2 : 1 AV block after revascularization; residual 1st-degree AV block (PR 220 ms).

Given the history of ischemic cardiomyopathy and symptoms consistent with unstable angina, a decision was made to proceed with left heart catheterization before pacemaker implantation. Coronary angiography revealed a dominant right coronary artery (RCA) with 80% ostial stenosis (Figure 2(a)). A drug-eluting stent was successfully deployed to the ostial RCA lesion with an outstanding angiographic result (Figure 2(b)). Upon revascularization, the patient immediately reverted to 1 : 1 AV conduction (Figure 3) and has remained in normal sinus rhythm with mild first-degree AV block since then. The lightheadedness and fatigue have entirely resolved. Permanent pacemaker implantation was avoided.

## 3. Theoretical Mechanisms of AV Block Reversibility in Coronary Artery Disease

The noteworthy finding in this case is the complete resolution of symptomatic high-grade AV block upon revascularization of the RCA. Review of the literature reveals two possible mechanisms for the presence of clinically significant AV block in patients without MI who experience restoration of normal AV conduction following coronary revascularization. These mechanisms are vagally mediated heart block and ischemia-driven conduction delay.

### 3.1. Vagal Hypothesis

Vagally mediated bradyarrhythmia is well documented in patients with myocardial ischemia or injury. Ischemic-mediated mechanical stretch and chemical substances stimulate receptors located in the inferior and posterior left ventricular walls [28]. These receptors lead to activation of nonmyelinated afferent C-fibers from the vagus nerve, which in turn result in increased vagal tone and bradyarrhythmia [28, 29]. This mechanism is known as the Bezold-Jarisch reflex. One case report discusses a patient with non-ST elevation MI who developed complete heart block in the setting of a 90% stenosis of the RCA acute marginal branch. The AV block resolved after balloon angioplasty of this lesion. Because the acute marginal branch does not supply the AV node, the mechanism for third-degree AV block was attributed to the Bezold-Jarisch reflex triggered by inferior wall ischemia [29].

Patients with CAD are especially susceptible to vagal stimulation, as demonstrated by the frequency of carotid sinus hypersensitivity in this population, as well as by the correlation of carotid hypersensitivity with the severity of CAD [30–32]. Jick and Linenthal, for example, reported a case of 2 : 1 AV block in an 85-year-old man with two previous myocardial infarctions and ischemic cardiomyopathy. In a time before percutaneous coronary intervention was available, they observed complete reversal of the 2 : 1 AV block to 1 : 1 AV conduction with atropine administration, as well as progression to complete heart block with carotid sinus massage, phenomena consistent with a vagal etiology of the conduction system disease [33]. Furthermore, coronary revascularization has been shown to attenuate postexercise heart rate decay in patients with RCA lesions, which suggests decreased vagal activity following reperfusion in inferior wall ischemia [34].

Although these descriptions confirm biologic plausibility, whether the decreased cardiac sensitivity to vagal stimuli after revascularization can fully reverse 2 : 1 AV block and avoid further episodes of high-grade AV block is difficult to prove and appears to be a rare phenomenon. Moreover, if the heart block was solely mediated by increased vagal tone, one would expect to see episodes of intermittent AV block during times of high vagal tone, despite revascularization.

### 3.2. Ischemia Hypothesis

AV conduction defects that resolve with revascularization may occur as a direct result of ischemia, a circumstance more consistent with complete postrevascularization restoration of 1 : 1 AV conduction, such as that observed in the case above. The AV node blood supply is provided by the AV nodal branch, which most commonly arises from the RCA [35], although it can rarely be a branch of the circumflex artery in patients with left coronary artery dominance [36–38]. Meanwhile, infranodal conduction system structures are supplied almost entirely by the septal perforator branches of the LAD artery, with variable dual supply provided by either the RCA or left circumflex artery [38–41]. Decreased flow to the septal branches or RCA is therefore associated with a variety of conduction disturbances [42, 43]. Importantly, the presence of high-grade AV block is associated with a 4-fold and 3-fold increased risk of in-hospital mortality for anterior and inferior wall acute infarctions, respectively [44]. Further, the presence of third-degree AV block in inferior MI has also been associated with an increased incidence of sustained hypotension and ventricular tachyarrhythmia [45].

In acute inferior wall MI, where the RCA is often the culprit, high-grade AV block has been described in up to 17% of cases [26]. Most of these cases are transient and resolve either spontaneously or with revascularization, whereas approximately 9% will ultimately require a permanent pacemaker, implicating permanent damage to AV conduction tissue prior to or due to lack of revascularization [46, 47]. The 2008 ACC/AHA/HRS Guidelines for Device-Based Therapy acknowledge that, in cases of third-degree AV block complicating inferior wall MI, permanent pacing should be reserved for patients in whom the block does not resolve with revascularization [1]. The possibility of transient AV block secondary to myocardial ischemia in patients without MI is not discussed in current practice guidelines [1].

### 3.3. AV Block Reversibility in Non-Myocardial Infarction Presentations

In patients with CAD presenting without acute MI, the reversibility of high-grade AV block and the avoidance of pacemaker implantation via revascularization are infrequently described in the literature. In a single-center retrospective study evaluating the reversibility of AV block in patients with CAD, Hwang et al. assessed 188 patients with high-grade AV block for the presence of concomitant CAD. Fifty-eight (30.8%) individuals were found to have CAD, distributed as follows: stable angina, 41; acute MI, 15; and unstable angina, 2. As expected, AV block was reversible with revascularization in 13 of the 15 patients presenting with acute MI. The culprit lesion was located in the RCA in 14 of the 15 acute MI patients. Conversely, only 1 of the 43 patients (2.3%) with stable angina and none of the 2 patients with unstable angina reverted to 1 : 1 AV conduction after revascularization, despite the fact that roughly 60% (26/43) of these patients also had significant RCA lesions [48].

Yesil et al. studied 53 pacemaker patients with complete heart block and significant CAD, defined by the presence of a coronary lesion with greater than 70% stenosis. In this study, patients with acute coronary syndrome were excluded. After a mean follow-up of 36 ± 6 months, third-degree AV block persisted in 13/16 (81%) patients treated medically and in 27/37 (73%) of the revascularized patients. Despite most of the lesions being in the RCA, the difference was not significant, leading the authors to conclude that, in the absence of acute MI, coronary revascularization has minimal impact on regaining normal AV function in patients with concomitant third-degree AV block and CAD [49].

The remaining published assessments of AV block in CAD patients without MI are smaller case series. Omeroglu et al., for example, reported a series of 8 patients who presented with new-onset complete AV block and severe CAD requiring coronary artery bypass grafting. Revascularization was performed on the same admission, but none of the patients had resolution of complete AV block in a follow-up of 10 ± 1.07 days [50]. Narin et al. reported two cases of complete heart block in patients with unstable angina that required CABG. When AV block in one of the patients remained on postoperative day 15, he received a permanent pacemaker. The other patient, however, reverted to 1 : 1 AV conduction immediately after surgical revascularization, and the absence of significant AV block remained at a follow-up of 27.7 months [51]. Such an outcome, we believe, is likely analogous to the fortuitous clinical course experienced by the patient we describe above.

## 4. Conclusion

In summary, while new-onset high-grade AV block in the setting of CAD is less likely to be reversible in patients without acute MI, there are patients in whom revascularization leads to immediate and durable resolution of 1 : 1 AV conduction. Examples of this phenomenon include the case illustration above as well as anecdotal cases in the literature. This clinical course is fairly unusual and the mechanism responsible is uncertain. Possible mechanisms include vagal mediation and, more likely, ischemia. The majority of published data suggest that high-grade AV block is usually not reversible with revascularization in patients who have CAD and do not present with an acute MI. The rare case such as the one we describe above is a fortunate but currently unpredictable exception to the rule that such patients will require pacemaker implantation. Nevertheless, in light of the potential negative impacts of permanent pacemakers, it may be prudent to observe postrevascularization conduction before committing patients to device implantation.
